# Supplementary material for: Evolutionary alterations in gene expression and enzymatic activities of gibberellin 3-oxidase 1 in Oryza
Source: Commun Biol. 2022 Jan 19;5:67. doi: 10.1038/s42003-022-03008-5 (PMC8770518; doi:10.1038/s42003-022-03008-5)
Supplement: Supplementary file 2 — Supplementary Information [file 42003_2022_3008_MOESM2_ESM.pdf]

Supplementary Information

Evolutionary alterations in gene expression and enzymatic activities of gibberellin 3-oxidase 1 in *Oryza*

Kyosuke Kawai[1], Sayaka Takehara[1], Toru Kashio[1], Minami Morii[1], Akihiko Sugihara[1], Hisako Yoshimura[1], Aya Ito[1], Masako Hattori[1], Yosuke Toda[2, 3], Mikiko Kojima[4], Yumiko Takebayashi[4], Hiroyasu Furuumi[5], Ken-ichi Nonomura[6], Bunzo Mikami[7], Takashi Akagi[8], Hitoshi Sakakibara[4, 9], Hidemi Kitano[1], Makoto Matsuoka[1], Miyako Ueguchi-Tanaka[1*]

* Corresponding author
email: mueguchi@nuagr1.agr.nagoya-u.ac.jp

[1]Bioscience and Biotechnology Center, Nagoya University, Chikusa, Nagoya 464-8601, Japan.
[2]Japan Science and Technology Agency, Kawaguchi, Saitama 332-0012, Japan.
[3]Institute of Transformative Bio-Molecules, Nagoya University, Chikusa, Nagoya 464-8602, Japan.
[4]RIKEN Center for Sustainable Resource Science, Tsurumi, Yokohama 230-0045, Japan.
[5]Technical Section, National Institute of Genetics, Mishima, Shizuoka 411-8540, Japan.
[6]Plant Cytogenetics, National Institute of Genetics, Mishima, Shizuoka 411-8540, Japan.
[7]Division of Applied Life Sciences, The Graduate School of Agriculture, Kyoto University, Uji 611-0011, Japan.
[8]Graduate School of Environmental and Life Science, Okayama University, Okayama 700-8530, Japan.
[9]Graduate School of Bioagricultural Sciences, Nagoya University, Nagoya, 464-8601, Japan.

Supplementary Tables

**Data collection**	
Beam line	SPring-8 BL26B1
Wavelength (Å)	1.0
Detector	MX225HE
Space group	P21
Molecules (Asymmetric Unit)	1
Cell dimensions (Å, °)	*a* = 56.84, *b* = 45.59, *c* = 68.54
	α = 90.00, β = 102.25, γ = 90.00
Resolution (Å) *	50.00–1.9 (1.93–1.9)
Total reflections	117157
Unique reflections	27238
Completeness (%) *	99.7 (95.7)
R_{merge} (%) *	4.7 (22.1)
Average <I>/<σ(I)> *	38.4 (7.8)
**Refinement statistics**	
Number of residues	354
Number of water molecules	296
R-$_{working}$/R_{free} (%)	16.51/20.82
R.m.s.d.bond length (Å)	0.010
R.m.s.d.bond angles (deg)	1.307

* Data for the highest resolution shell are listed in parentheses.

Supplementary Table 1. Data collection and refinement statistics[1-7] of OsGA3ox2.

Plant species	Gene name in this study	Gene ID	Data Source*
Oryza sativa	*Oryza_sativa_GA3ox1, OsGA3ox1*	LOC_Os05g08540	TIGR
	Oryza_sativa_GA3ox2, OsGA3ox2	LOC_Os01g08220	
Oryza nivara	*Oryza_nivara_GA3ox1*	ONIVA05G05590	EnsemblPlants
	Oryza_nivara_GA3ox2	ONIVA01G05670	
Oryza rufipogon	*Oryza_rufipogon_GA3ox1*	ORUFI05G05470	EnsemblPlants
	Oryza_rufipogon_GA3ox2	ORUFI01G05300	
Oryza barthii	*Oryza_barthii_GA3ox1*	OBART05G05200	EnsemblPlants
	Oryza_barthii_GA3ox2	Not annotated	
Oryza meridionalis	*Oryza_meridionalis_GA3ox1*	OMERI07G10700	EnsemblPlants
	Oryza_meridionalis_GA3ox2	OMERI01G05010	
Oryza glumipatula	*Oryza_glumipatula_GA3ox1*	OGLUM05G05060	EnsemblPlants
	Oryza_glumipatula_GA3ox2	OGLUM01G05460	
Oryza punctata	*Oryza_punctata_GA3ox1*	OPUNC05G04790	EnsemblPlants
	Oryza_punctata_GA3ox2	OPUNC01G04850	
Oryza brachyantha (W0654)	*Oryza_brachyantha_GA3ox1*		This study
	Oryza_brachyantha_GA3ox2		
Oryza granulata (W0008)	*Oryza_granulata_GA3ox1*		This study
	Oryza_granulata_GA3ox2		
Leersia perrieri (W1529)	*Leersia_perrieri_GA3ox1*	LPERR05G4620	EnsemblPlants
	Leersia_perrieri_GA3ox2	Not annotated	
Hordeum vulgare	*Hordeum_vulgare_GA3ox1*	AY551430	NCBI
	Hordeum_vulgare_GA3ox2	AY551431	
Triticum aestivum	*Triticum_aestivum_GA3ox-2A*	TraesCS2A02G540400	EnsemblPlants
	Triticum_aestivum_GA3ox-2B	TraesCS2B02G570900	
	Triticum_aestivum_GA3ox-2D	TraesCS2D02G542100	EnsemblPlants
	Triticum_aestivum_GA3ox-3A	TraesCS3A02G122600	
	Triticum_aestivum_GA3ox-3B	TraesCS3B02G141800	EnsemblPlants
	Triticum_aestivum_GA3ox-3D	TraesCS3D02G124500	
Brachypodium distachyon	*Brachypodium_distachyon_GA3ox2a*	Bradi2g04840(a)	EnsemblPlants
	Brachypodium_distachyon_GA3ox2b	Bradi4g23570(b)	
Oropetium thomaeum	*Oropetium_thomaeum_Oropetium_20150105_07284A*	Oropetium_20150105_07284A	phytozome
Sorghum bicolor	*Sorghum_bicolor_GA3ox1*	Sobic.009G064700	phytozome
	Sorghum_bicolor_GA3ox2	Sobic.003G045900	
Zea mays	*Zea_mays_GA3ox1*	Zm00001d037627_T001	EnsemblPlants
	Zea_mays_GA3ox2	Zm00001d039634_T001	
Setaria italica	*Setaria_italica_Si025127m.g*	Si025127m.g	EnsemblPlants
	Setaria_italica_Si001832m.g	Si001832m.g	
Panicum virgatum	*Panicum_virgatum_Pavir.J24392.1*	Pavir.J24392.1	phytozome
	Panicum_virgatum_Pavir.Eb00358.1	Pavir.Eb00358.1	
	Panicum_virgatum_Pavir.J17485.1	Pavir.J17485.1	
Ananas comosus	*Ananas_comosus_Aco009790.1*	Aco009790.1	phytozome
	Ananas_comosus_Aco014326.1	Aco014326.1	
Musa acuminata	*Musa_acuminata_GA3ox1*	GSMUA_Achr1T03100_001	EnsemblPlants
	Musa_acuminata_GA3ox2	GSMUA_Achr4T08970_001	
	Musa_acuminata_GA3ox3	GSMUA_Achr5T09790_001	
	Musa_acuminata_GA3ox4	GSMUA_Achr7T13240_001	
	Musa_acuminata_GA3ox5	GSMUA_AchrUn_randomT03870_001	
Spirodela	*Spirodela_polyrhiza_Spipo21G0010100*	Spipo21G0010100 (primary)	phytozome

polyrhiza	*Spirodela_polyrhiza_Spipo21G0010200*	Spipo21G0010200 (primary)	
Zostera marina	*Zostera_marina_Zosma208g00280.1*	Zosma208g00280.1	phytozome
Arabidopsis thaliana	*Arabidopsis_thaliana_GA3ox1*	AT1G15550	TAIR
	Arabidopsis_thaliana_GA3ox2	AT1G80340	
	Arabidopsis_thaliana_GA3ox3	AT4G21690	
	Arabidopsis_thaliana_GA3ox4	AT1G80330	
Glycine max	*Glycine_max_Glyma.04G071000*	Glyma.04G071000	phytozome
	Glycine_max_Glyma.06G072600	Glyma.06G072600	
	Glycine_max_Glyma.07G033800	Glyma.07G033800	
	Glycine_max_Glyma.08G208300	Glyma.08G208300	
	Glycine_max_Glyma.13G361700	Glyma.13G361700	
	Glycine_max_Glyma.14G128400	Glyma.14G128400	
	Glycine_max_Glyma.15G012100	Glyma.15G012100	
	Glycine_max_Glyma.17G205300	Glyma.17G205300	
Solanum lycopersicum	*Solanum_lycopersicum_GA3ox1*	Solyc06g066820	phytozome
	Solanum_lycopersicum_GA3ox2	Solyc03g119910	
	Solanum_lycopersicum_GA3ox3	Solyc01g058250	
	Solanum_lycopersicum_GA3ox-like-1	Solyc00g007180	
	Solanum_lycopersicum_GA3ox-like-2	Solyc05g052740	
Vitis vinifera	*Vitis_vinifera_GA3ox1*	GSVIVT01008811001	phytozome
	Vitis_vinifera_GA3ox2	GSVIVT01017173001	
	Vitis_vinifera_GA3ox3	GSVIVT01017178001	
	Vitis_vinifera_GA3ox4	GSVIVT01020680001	
	Vitis_vinifera_GA3ox5	GSVIVT01026928001	
	Vitis_vinifera_GA3ox6	GSVIVT01035796001	
Amborella trichopoda	*Amborella_trichopoda_evm_27.model.AmTr_v1.0_scaffold00122.35*	evm_27.model.AmTr_v1.0_scaffold00122.35 (primary)	phytozome
Pinus tabuliformis	*Pinus_tabuliformis_AHW42463*	AHW42463	NCBI
	Pinus_tabuliformis_AHW42464	AHW42464	
Picea abies	*Picea_abies_MA_10249g0010*	MA_10249g0010	ConGenIE
	Picea_abies_MA_24155g0010	MA_24155g0010	
Lygodium japonicum	*Lygodium_japonicum_Isotig12104*	Isotig12104	Lygodium japonicum Transcriptome Database
	Lygodium_japonicum_Isotig09392	Isotig09392	
Selaginella moellendorffii	*Selaginella_moellendorffii_GA3ox*	446928 (primary)	phytozome
Physcomitrella patens	*Physcomitrella_patens_GA3oxL*	Pp3c17_7090V3.1 (primary)	phytozome
Oryza sativa	*Oryza_sativa_GA20ox2, OsGA20ox2*	LOC_Os01g66100	TIGR
	OsGA20ox1	LOC_Os03g63970.1	
	OsGA20ox3	LOC_Os07g07420.1	
	OsGA20ox4	LOC_Os05g34854.1	
	OsGA2ox1	LOC_Os05g06670.1	
	OsGA2ox2	LOC_Os01g22910	
	OsGA2ox3	LOC_Os01g55240	
	OsGA2ox4	LOC_Os05g43880	
	OsGA2ox5	LOC_Os07g01340	
	OsGA2ox6	LOC_Os04g44150	
	OsGA2ox7	LOC_Os01g11150	
	OsGA2ox8	LOC_Os05g48700	
	OsGA2ox9	LOC_Os02g41954.1	
	OsGA2ox10	LOC_Os05g11810.1	

*TIGR, The Institute for Genomic Research Rice Genome Annotation project (http://rice.plantbiology.msu.edu/); EnsemblPlants (http://plants.ensembl.org/index.html); Phytozome (https://phytozome.jgi.doe.gov/pz/portal.html); NCBI (https://blast.ncbi.nlm.nih.gov/Blast.cgi); Lygodium japonicum Transcriptome Database (http://bioinf.mind.meiji.ac.jp/kanikusa/); ConGenIE (http://congenie.org); TAIR The Arabidopsis Information Resource (http://www.arabidopsis.org/index.jsp).

Supplementary Table 2. GA3ox orthologs of land plants used in this study.

Experiments	Sequence (5'-3')	Description
Cas9 target	GTTGACATCTGCTTCGGGTACCGG	Cas9 target (guide RNA)
	AAACCCGGTACCCGAAGCAGATGT	
Genotyping	ATCACGCGAAAGAAATGGTC	For *OsGA3ox1*
	ATGATGGGTGCAGTGCTATG	
	AACTCACGACAAAAATGCTT	For *O.brachyanthaGA3ox1*
	TGCAGGATGAAGGTGAAGAA	
	ATCTCGCGGCAAAAACGCTC	For *O.granulataGA3ox1*
	GGTGTGTCGACGTCACTGAG	
	CCCGCAAGTCTGAAGAAACT	For Cas9
	ATACCTGGGCCTTTCTGGAT	
GUS transgenic	cctctagaCGTACACACCTGACAACAAC	For *GUS*
	CCcccgggCTTGAAGAACATGGCGAGG	
	GCTTCTGCTGCTACGAGACC	For *GA3ox1* gene cloning of *Oryza GA3ox1s* and *L.perrieriGA3ox1*
	AGGTCCTCTTCTCCGAGCAC	
	TATTGGGTACTACTAAAGCTTAACGAATTCGCCCTT	For In-fusion
	GGGAAATTCGAGCTCGGTACCCGCGAATTCGCCCTT	
5'/3' RACE	CATGTGGTCCGAGGGCTACACC	For sequencing of *Oryza GA3ox1*s and *L.perieriGA3ox2*
	AGACGCTGTGGAAGCGGCCGTT	
	CTAATACGACTCACTATAGGGCAAGCAGTGGTATCAACGCAGAGT	For sequencing of *O.brachyanthaGA3ox1* and *O.granulataGA3ox1*
	CTAATACGACTCACTATAGGGC	
	GTAAAACGACGGCCAG	For sequencing of pCR4 Blunt-TOPO vector
	CAGGAAACAGCTATGAC	
qRT-PCR target : *GA3ox1*	TCAACATGTTCCCTAGGTGTCC	For *O.sativaGA3ox1* and *O.rufipogonGA3ox1*
	GAAGGTGAAGAAGCCTGAGTCC	
	ACGATTCATCTGAACATGTT	For *O.punctataGA3ox1*
	GAAGGTGAAGAAGCCCGAGT	
	GCTGCTCGCCATGTTCTT	For *O.australiensisGA3ox1*
	ATCCGGACACCTGGGAAACAT	
	GAGAGGCTGCTCTCCATGTT	For *O.brachyanthaGA3ox1*
	TCCGGACACCTCGGAAACAG	
	GACTACCACCGCTTCTGCTC	For *O.granulataGA3ox1*
	GTCGAGCGCCTTGAAGAAC	
	GACTACCACCGCTTCTGCTC	For *L.perrieriGA3ox1*
	CTTGGGGTACATGTTCAGGT	
	GCCGGTGGTGGACATGAG	For *O.sativaGA3ox1* / *osga3ox1* and *O.rufipogonGA3ox1* / *osga3ox1*
	CCCACATTTGTTTCGGGTACC	
	GCCGGTGGTGGACATGAG	For *O.brachyanthaGA3ox1* / *osga3ox1*
	ACATGCGCTTCGGGTACC	
	GCCGGTGGTGGACATGCG	For *O.granulataGA3ox1* / *osga3ox1*
	ACATGCGCTTCGGGTAGC	
	GCCGGTCGTCGACATGCG	For *L.perrieriGA3ox1* / *osga3ox1*
	CACATCCGCTTCGGGTACC	
qRT-PCR target : *OsCIN3*	GCTTCAAGTCCGTGCAGTCG	For *OsCIN3*
	CCGAGCTTCTTGCACAGC	
qRT-PCR target : internal control (*UBQ5* gene)	AGCACAAGCACAAGAAGGTGAAG	For *UBQ5s* of *O.sativa*, *O.rufipogon* and *O.punctata*
	AGTAGTGGCGGTCGAAGTGGTT	
	CGCCGACTACAACATCCAG	For *O.australiensisUBQ5*
	CTTCTTCTTGCGCTTCTTGG	
	CTACACCAAGCCCAAGAAGC	For *O.brachyantha*UBQ5
	GCGTCGTCGACCTTGTAGAAC	
	CTACACCAAGCCGAAGAAGC	For *O.granulata*UBQ5
	GCGTCGTCAACCTTGTAGAAC	
	ACCCTCGCCGACTACAACAT	For *L.perrieriUBQ5*
	CTCCTTCCTGAGGCGAGTAA	
Expression and prufication of OsGA3ox2 and OsGA3ox1	CCGGATCCATGACATCGTCG	For *GST-OsGA3ox1*
	CCCCCGGGCTAACTCTCCTTG	
	CCGGATCCATGCCGACGC	For *GST-OsGA3ox2*
	CCGAATTCTTATGCGTGGACG	
	AACATGTACCCTAGGTGTCCAGATCCA	For mutagenize *GST-OsGA3ox1*
	CCTAGGGTACATGTTGAGGTGAATCGT	

Supplementary Table 3. Primer and guide RNA list used in this study.

Supplementary Figures

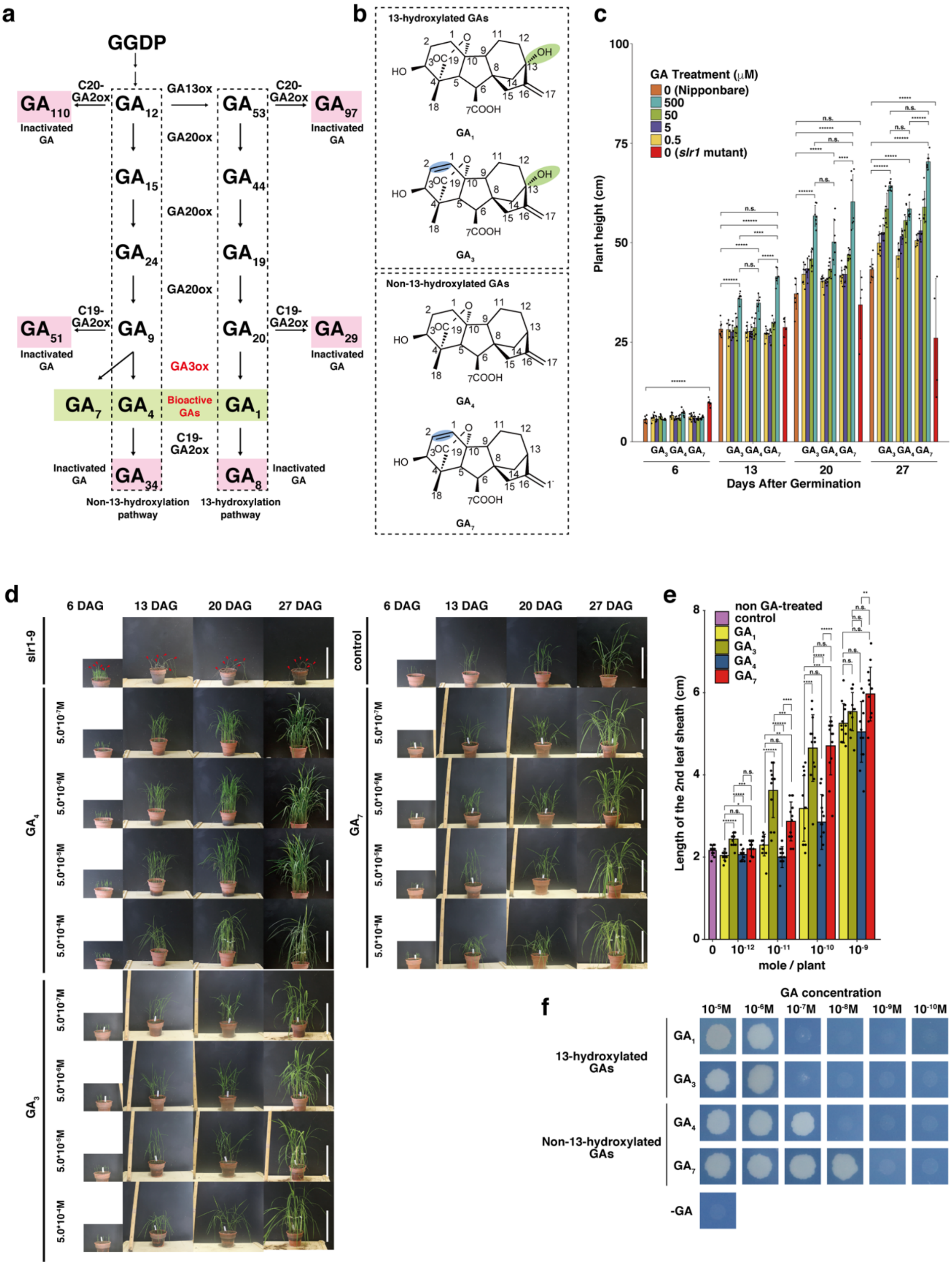

Supplementary Figure 1. Gibberellic acid synthesis pathway and its bioactivities. a) The GA synthesis pathway. Rice possesses the GA 13- and non-13-hydroxylation pathways, and GA13ox adds -OH to C13 of GA precursors. GAs are converted to the active form by GA3ox in the final step of GA

synthesis. C20-GA2oxs inactivate upstream GA precursors, and C19-GA2oxs inactivate bioactive GAs (GA_1 and GA_4) and the direct precursors (GA_9 and GA_{20}). **b)** The structure of bioactive GAs. GA_1 and GA_3 are 13-hydroxylated GAs that have undergone 13-hydroxylation (circled in green). GA_4 and GA_7 are non-13-hydroxylated, without 13-hydroxylation. Since GA_3 and GA_7 possess a double bond between the C1 and C2 atoms (circled in blue), they are not inactivated by GA2ox. **c)** Plant heights of GA_1, GA_3, and GA_7 treated Nipponbare and *slr1* mutant. For the *slr* mutant, homozygous individuals were selected at 13 DAGs, and only these were measured. ****: $p < 0.00001$, *****: $p < 0.000001$, ******: $p < 0.0000001$, n.s.: not significant. n = 7, (only in *slr* mutant, n = 6 (6 and 13 DAG) or 4 (20 and 27 DAG) because of withering). All p-values are listed in Supplementary Data 2 **d)** Plant of GA_1, GA_3, and GA_7 treated Nipponbare and *slr1* mutant. Red arrowheads in *slr* mutant plants indicate homozygous mutants. The *slr1* mutant had withered for about 13 days. bars = 30 cm. **e)** Induction on second sheath length of Tan-Ginbozu seedlings by bioactive GAs (n = 13 for each bioactive GA). Error bars = s.d. ****: $p < 0.00001$, *****: $p < 0.000001$, ******: $p < 0.0000001$, n.s.: not significant. **f)** Analysis of GID1 and SLR1 binding at various concentrations of GA by using yeast two-hybrid systems. Yeast strains were grown on -His plates for 7 days.

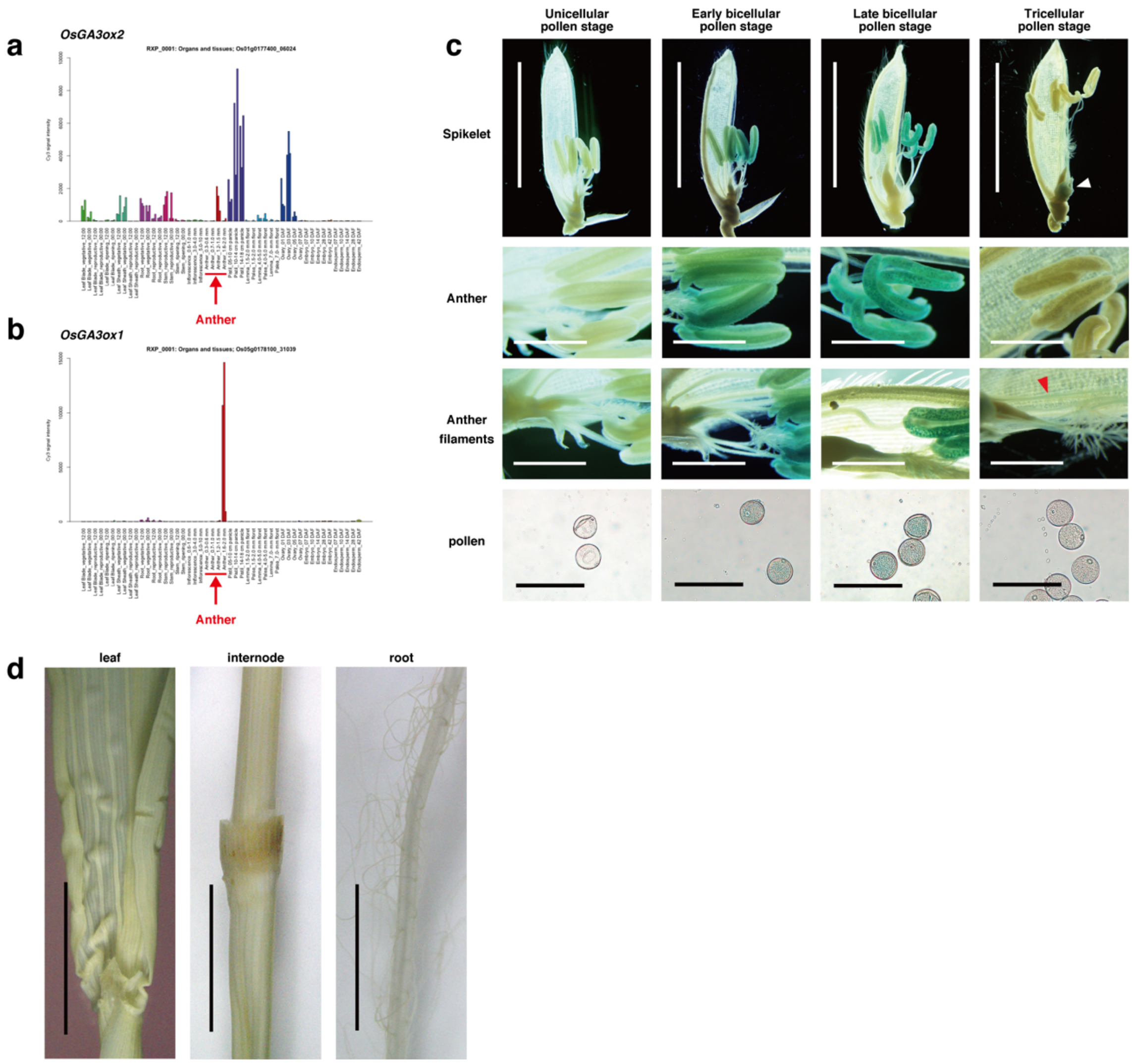

Supplementary Figure 2. Expression profiles of *OsGA3ox1* and *OsGA3ox2*. a and **b)** Tissue- and organ-specific expression profiles of *OsGA3ox2* (LOC_Os01g08220) (**a**) and *OsGA3ox1* (LOC_Os05g08540) (**b**) in rice based on data obtained from the RiceXpro database (https://ricexpro.dna.affrc.go.jp). **c)** GUS expression analysis. *OsGA3ox1* promoter-GUS was introduced into Nipponbare plants. Anther filament is indicated using a red arrowhead, and lodicules is depicted using a white arrowhead. Bars = 5 mm (spikelets), 1 mm (anthers), and 100 µm (pollen grains). **d)** GUS activity in vegetative organs with the same transformants as that observed in **c**. No GUS activity was observed in vegetative growth organs. Bars = 5 cm (leaf, internode, and root).

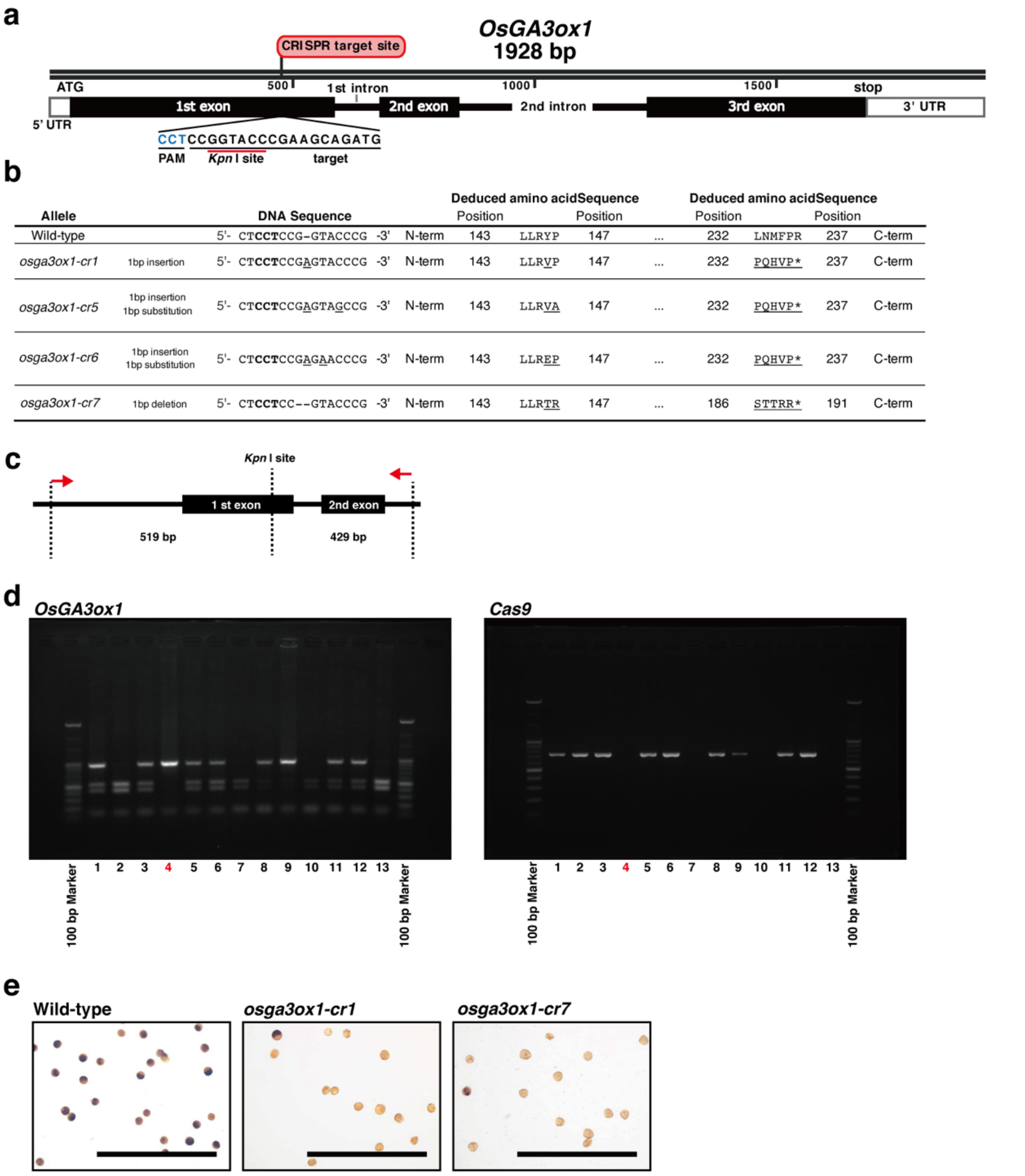

Supplementary Figure 3. Generation of *osga3ox1* knockout mutants by using CRISPR/Cas9. a) Diagram of the CRISPR/Cas9-mediated mutation introduced in *OsGA3ox1*. The white boxes, black boxes, and lines indicate the UTRs, exons, and introns, respectively. **b)** Modification of *OsGA3ox1* sequence conducted by using CRISPR/Cas9 in the T_0 generation. **c)** Schematic of genotyping of *osga3ox1* mutants analyzed by PCR and *Kpn*I digestion. The size of the PCR product is 948 bp, which is digested into two fragments of 519 and 429 bp in Nipponbare (wild type) but remains intact in the case of the *osga3ox1* mutant. Gene sequences of the *osga3ox1* mutants were confirmed by sequencing. **d)** Genotyping of *osga3ox1* and its internal *Cas9* using gDNA from leaf blades of T_1 individuals; those with the *osga3ox1* mutation and *Cas9* missing from the genome are indicated in red letters. **e)**

Phenotypic analysis by KI staining of pollen from different alleles of *osga3ox1* mutant. Bars = 0.5 mm.

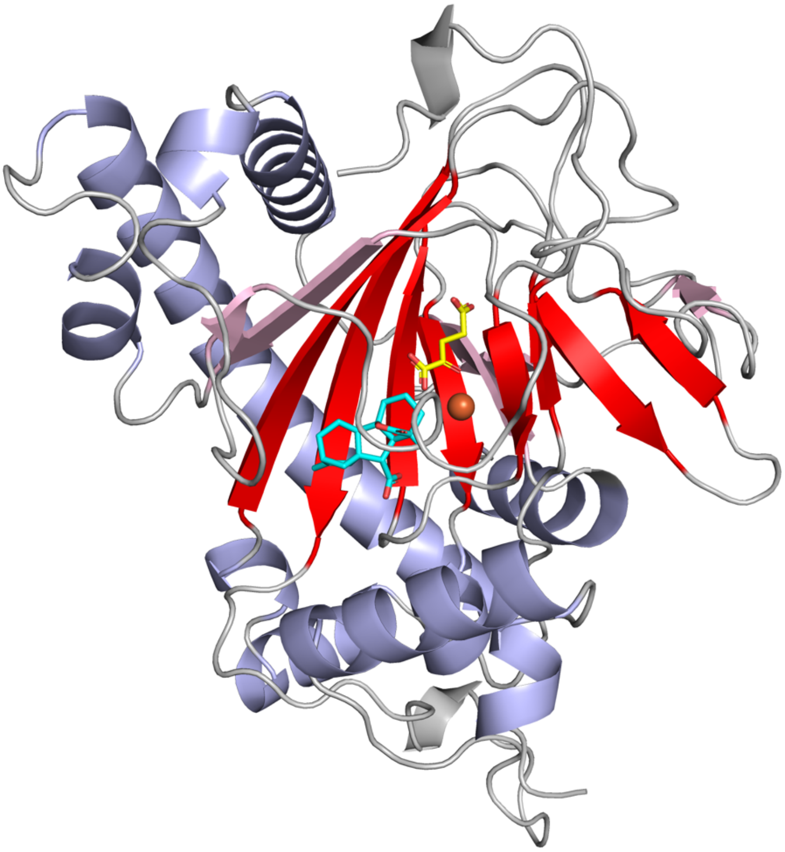

Supplementary Figure 4. Structure of OsGA3ox2 with substrates. Crystal structure of OsGA3ox2. The α-helices are shown in blue, the common core fold is shown in red, and non core β-strands are shown in pink. 2OG and GA_9 are shown as sticks and colored by atom with carbons in yellow and cyan, respectively. Fe (II) is colored in orange.

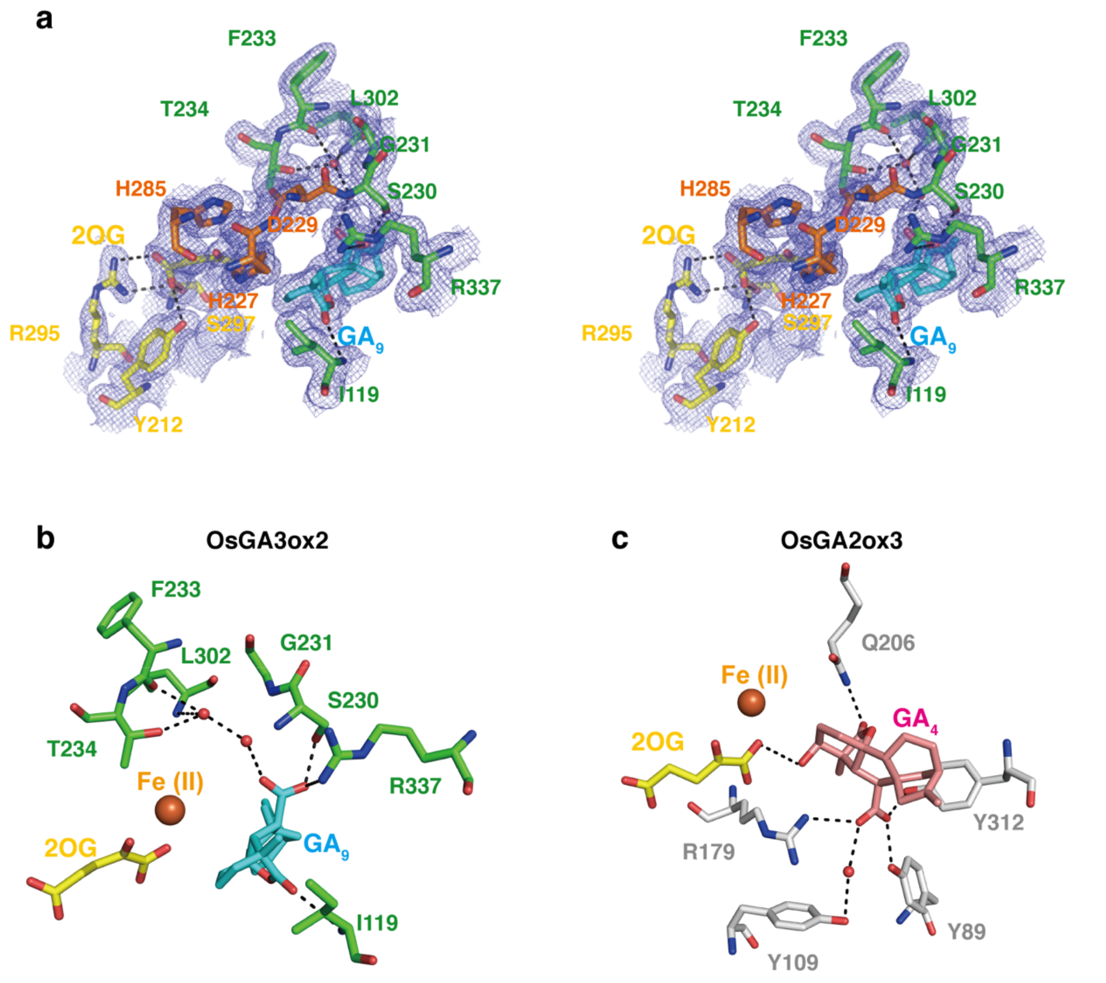

Supplementary Figure 5. Stereo views of the OsGA3ox2 active site and structural comparison of the active sites of OsGA3ox2 and OsGA2ox3. a) 2Fo-Fc electron density map contoured at 1.0 σ. The amino acids establishing interactions with Fe (II), 2OG, and GA_9 are indicated in orange, yellow, and green, respectively. The water molecules and hydrogen bonds are indicated by using red spheres and dashed lines, respectively. **b** and **c)** Close-up view of the structure of OsGA3ox2 (**b**) and OsGA2ox3 (**c**)[8] around the GA-binding site with GA_9 (blue, OsGA3ox2) and GA_4 (pink, OsGA2ox3) and co-substrate, Fe (II) (orange), and 2OG (yellow). The amino acids establishing interactions with GA_9 in OsGA3ox2 (I119, S230, G231, F233, T234, L302, and R337) and GA_4 in OsGA2ox3 (Y89, Y109, R179, Q206, and Y312) are indicated in green and white, respectively.

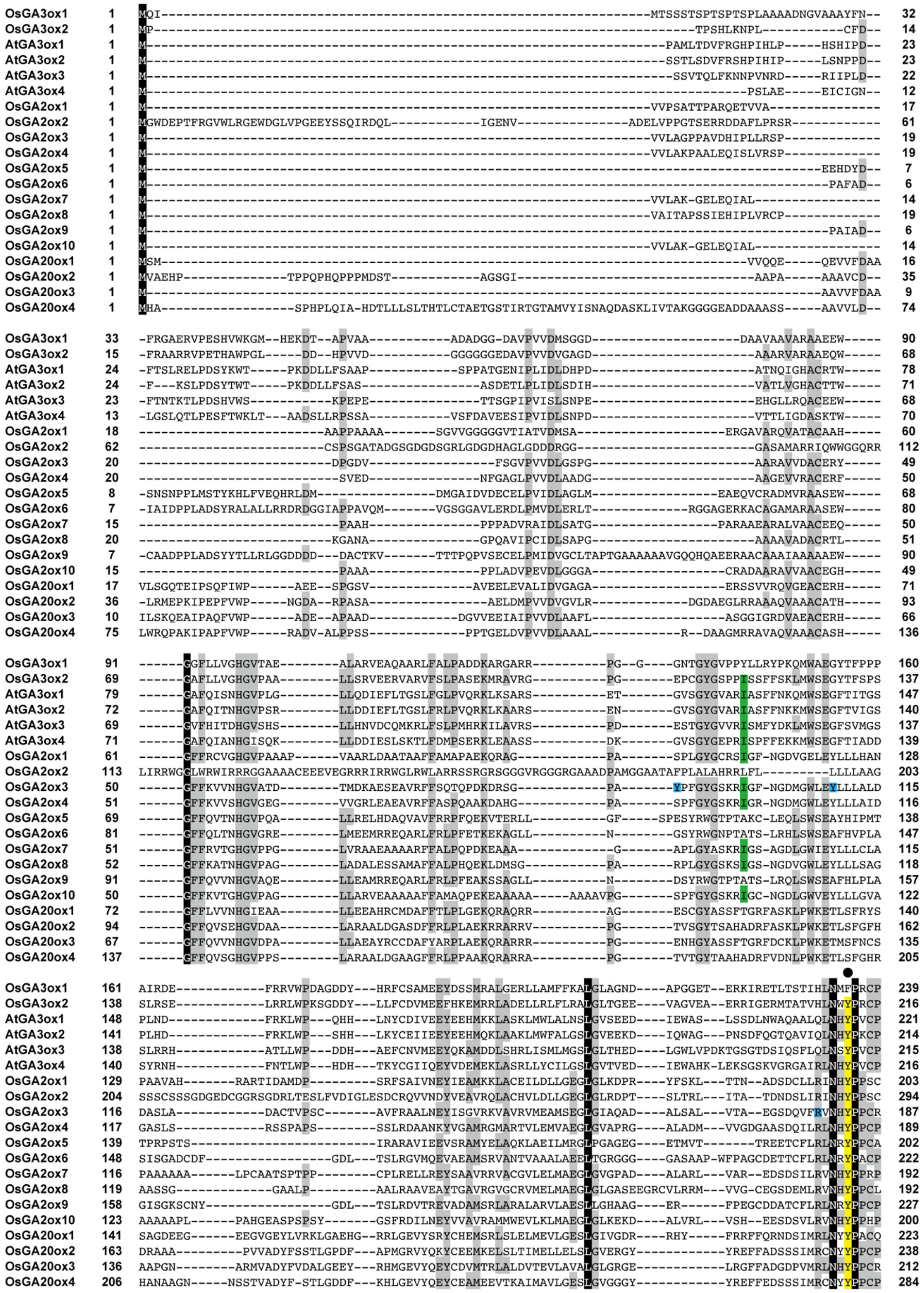

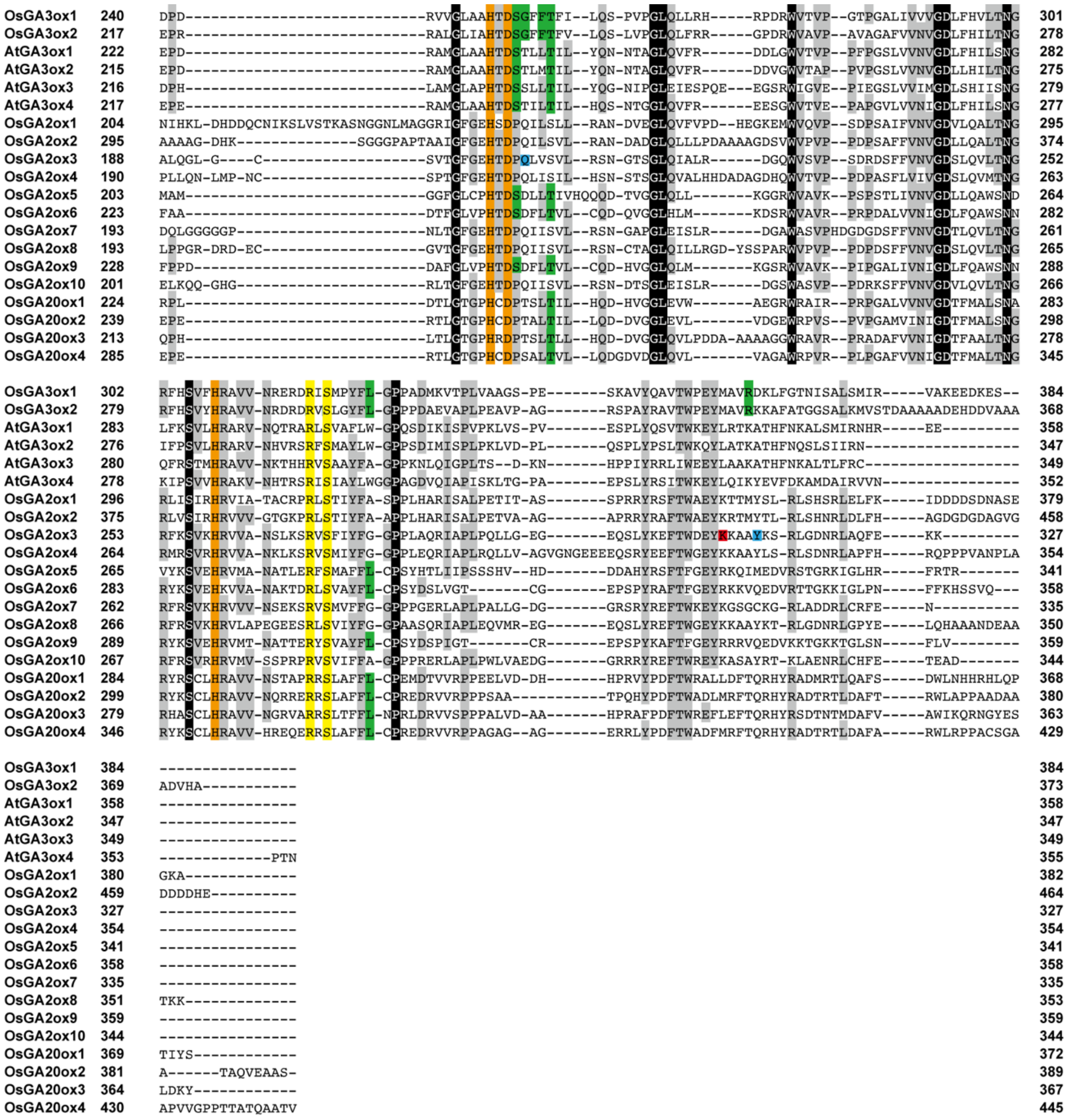

Supplementary Figure 6. Amino acid sequence alignment of OsGA3oxs AtGA3oxs, OsGA2oxs, and OsGA20oxs. Protein sequence data of OsGA3oxs, AtGA3oxs, OsGA2oxs, and OsGA20oxs collected from public databases (Supplementary Table 2) were aligned using MAFFT version 7.3 with the L-INS-i model[9]. Alignments are displayed using the GENETYX-MAC software (https://www.genetyx.co.jp). Residues with 100% identity are shown in black, and those with more than 50% identity are depicted in grey. The residues establishing interactions with Fe (II) and 2OG are highlighted in orange and yellow, respectively. Green-highlighted residues indicate the conserved residues that were determined by considering the crystal structure of OsGA3ox2 for binding to substrate GA_9. Regarding OsGA2ox3, the residues highlighted in blue and red represent the residues binding to GA_4 in the active site and intermolecular binding site, respectively, as shown previously by

Takehara et al.[8]. Species abbreviations: Os, *Oryza sativa*; At, *Arabidopsis thaliana*.

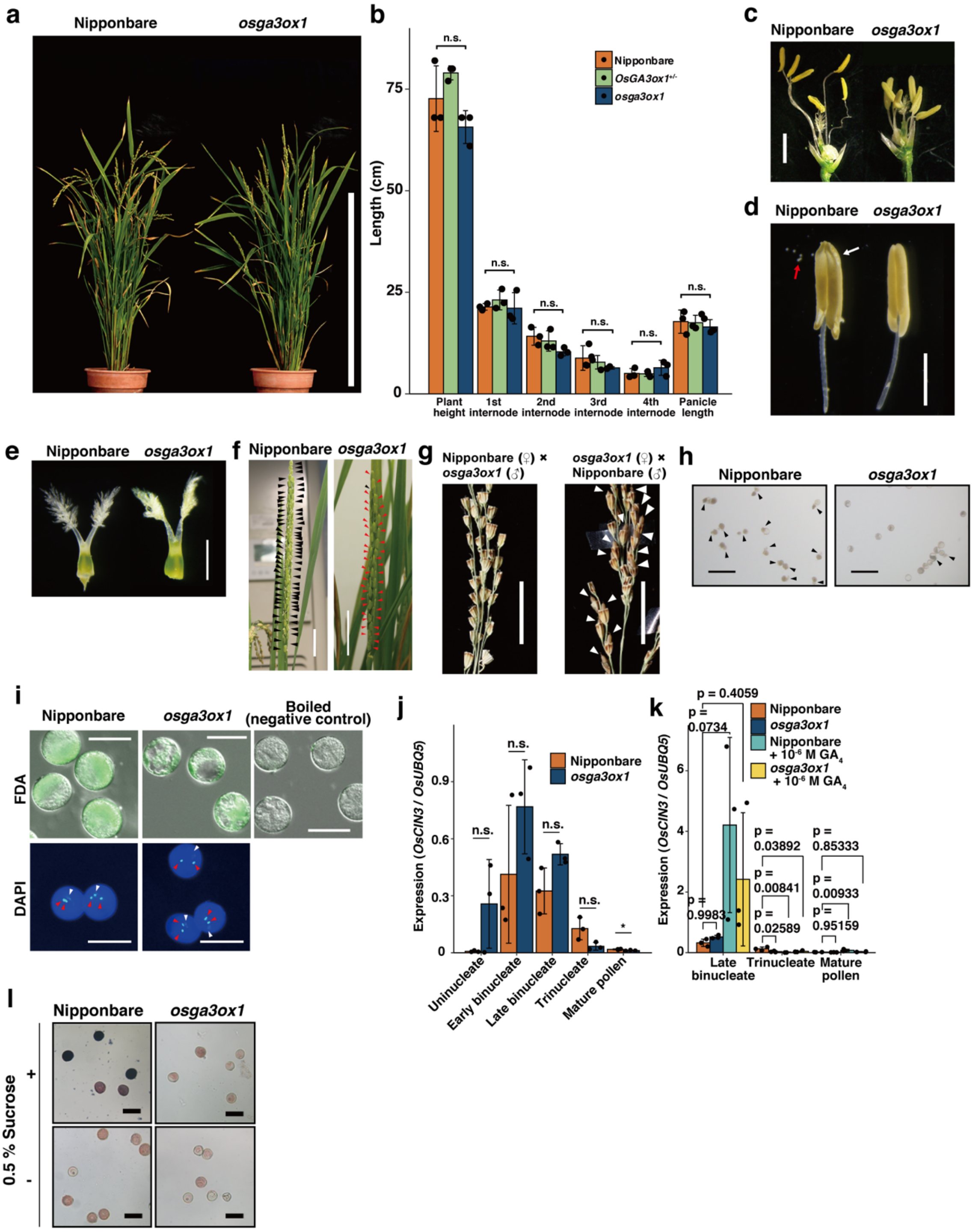

Supplementary Figure 7. ***osga3ox1*** **knockout rice are physiologically abnormal in the flowering stage. a)** Vegetative phenotype of Nipponbare (left) and *osga3ox1* knockout mutant (right). Bar = 50 cm. **b)** Evaluation of aboveground phenotype of Nipponbare, *OsGA3ox1*$^{+/-}$ (heterozygous), and *osga3ox1* mutant (homozygous). Error bars, s.d. n = 3, one-way ANOVA. n.s.; not significant ($p > 0.05$). **c-e)** Phenotype of the flowering spikelets with palea and lemma removed (**c**), anthers (**d**), and pistils (**e**) of Nipponbare and *osga3ox1* mutant. Red and white arrows indicate pollen grains and anther

dehiscence, respectively in **d**. Bars = 2 mm (**c**), 1 mm (**d** and **e**). **f)** Observation of flowering of Nipponbare and *osga3ox1* mutant panicles. Black and red arrowheads indicate opened and non-opened spikelets, respectively. Bars = 2 cm. **g)** Seed fertility of pistils upon reciprocal crossing test of Nipponbare pistils with *osga3ox1* mutant pollen (left) and *osga3ox1* mutant pistils with Nipponbare pollen (right) as described in Figure 2**f**. White arrowheads indicate seed setting. Bars = 2 cm. **h)** *In vitro* pollen tube germination of Nipponbare and *osga3ox1* mutant. Black arrowheads indicate pollen tube germination. bars = 100 μm. **i)** Staining of pollen grains in Nipponbare and *osga3ox1* mutant using FDA and DAPI for cell viability. Boiled pollen grains were used as a negative control ("Boiled"). Both wild-type and *osga3ox1* mutant pollen grains showed green fluorescence, indicating that the cells were alive. Red and white arrowheads indicate sperm nuclei and vegetative nucleus, respectively. Bars = 50 μm. **j** and **k)** qRT-PCR expression analysis of *cell-wall invertase 3* (*OsCIN3*) in anthers of Nipponbare and *osga3ox1* mutant (**j**) or those treated with GA_4 (**j**). Error bars, s.d. n = 3, two-tailed paired *t*-tests (***$p < 0.001$, **$p < 0.01$, *$p < 0.05$) (**i**), and p-values by Dunnett's multiple comparison test (**k**) are indicated. **l**) *In situ* stains of acid invertase activity of the Nipponbare and *osga3ox1* mutant pollen grains using nitroblue tetrazolium. Upper panels: + sucrose; lower panels: control (− sucrose). Pollen grains stained black possess invertase activity. Bars = 50 μm.

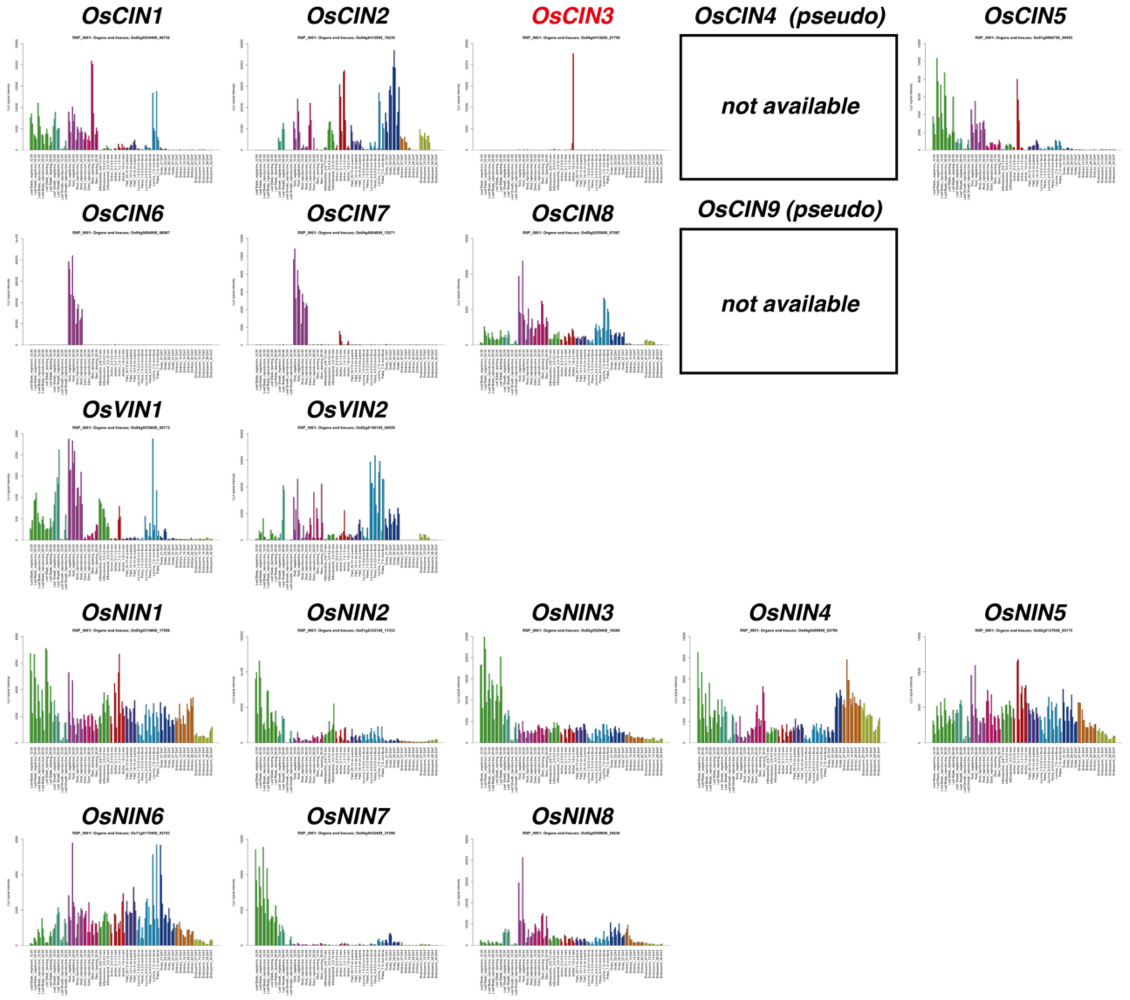

Supplementary Figure 8. Expression profiles of rice invertases. The tissue- and organ-specific expression profiles of nine *cell wall invertases* (*CINs*), eight *neutral invertases* (*NINs*), and two *vacuolar invertases* (*VINs*) obtained from the RiceXpro database (https://ricexpro.dna.affrc.go.jp). *OsCIN4* and *OsCIN9* are pseudogenes[10,11].

a

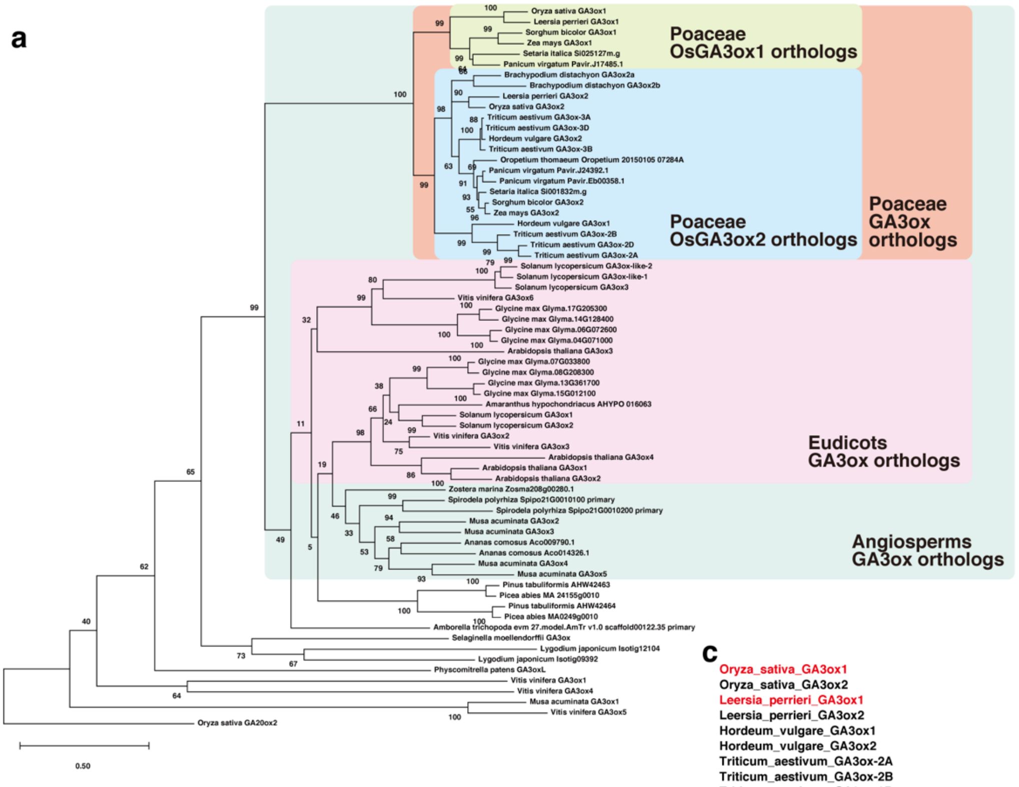

b

Clade	Subfamily	Tribe	Species	Copy number of orthologs GA3ox1	Copy number of orthologs GA3ox2
BOP clade	Ehrhartoideae	Oryzeae	*Oryza sativa*	1	1
			Leersia perrieri	1	1
	Pooideae	Triticeae	*Hordeum vulgare*	0	1 (+1)*1
			Triticum aestivum (allohexaploid)	0	6 (+1)*2
		Brachypodieae	*Brachypodium distachyon*	0	2
PACMAD clade	Chloridoideae	Cynodonteae	*Oropetium thomaeum*	0	1
	Panicoideae	Andropogoneae	*Sorghum bicolor*	1	1
			Zea mays	1	1
		Paniceae	*Setaria italica*	1	1
			Panicum virgatum	1	2

c

Oryza_sativa_GA3ox1	NMFPR
Oryza_sativa_GA3ox2	NWYPR
Leersia_perrieri_GA3ox1	NMYPK
Leersia_perrieri_GA3ox2	NWYPK
Hordeum_vulgare_GA3ox1	NWYPK
Hordeum_vulgare_GA3ox2	NWYPK
Triticum_aestivum_GA3ox-2A	NWYPK
Triticum_aestivum_GA3ox-2B	NWYPK
Triticum_aestivum_GA3ox-2D	NWYPK
Triticum_aestivum_GA3ox-3A	NWYPK
Triticum_aestivum_GA3ox-3B	NWYPK
Triticum_aestivum_GA3ox-3D	NWYPK
Brachypodium_distachyon_GA3ox2a	NWYPR
Brachypodium_distachyon_GA3ox2b	NWYPK
Oropetium_thomaeum_Oropetium_20150105_07284A	--YPR
Sorghum_bicolor_GA3ox1	ILYPK
Sorghum_bicolor_GA3ox2	NWYPR
Zea_mays_GA3ox1	ILYPR
Zea_mays_GA3ox2	NWYPR
Setaria_italica_Si025127m.g	NMYPR
Setaria_italica_Si001832m.g	NWYPR
Panicum_virgatum_Pavir.J24392.1	NWYPR
Panicum_virgatum_Pavir.Eb00358.1	NWYPR
Panicum_virgatum_Pavir.J17485.1	NLYPR
Oryza_sativa_GA20ox2	NYYPP

Supplementary Figure 9. Phylogenetic relationship among orthologs of GA3ox of land plants and GA3ox orthologs in Poaceae plants. a) Phylogenetic tree of GA3ox orthologs of land plants constructed using Bayesian analysis with the ML method using a JTT + I + G substitution model generated by MEGA version 10. **b)** Phylogenetic relationships of Poaceae species[12] and copy number of GA3ox orthologs. **c)** Amino acid sequence alignment around F in the 2OG-interacting site of GA3ox orthologs in land plants. Residues of F in the interacting site of 2OG are shown in green, and those with more than 50% identity are indicated in grey. Genes indicated in red letters represent OsGA3ox1 orthologs.*1: one of the orthologs possesses GA1-oxidase activity and has been designated as GA1ox-B1[13]; *2: one of them is bifunctional and has been designated as "HvGA3, 18ox1"[13].

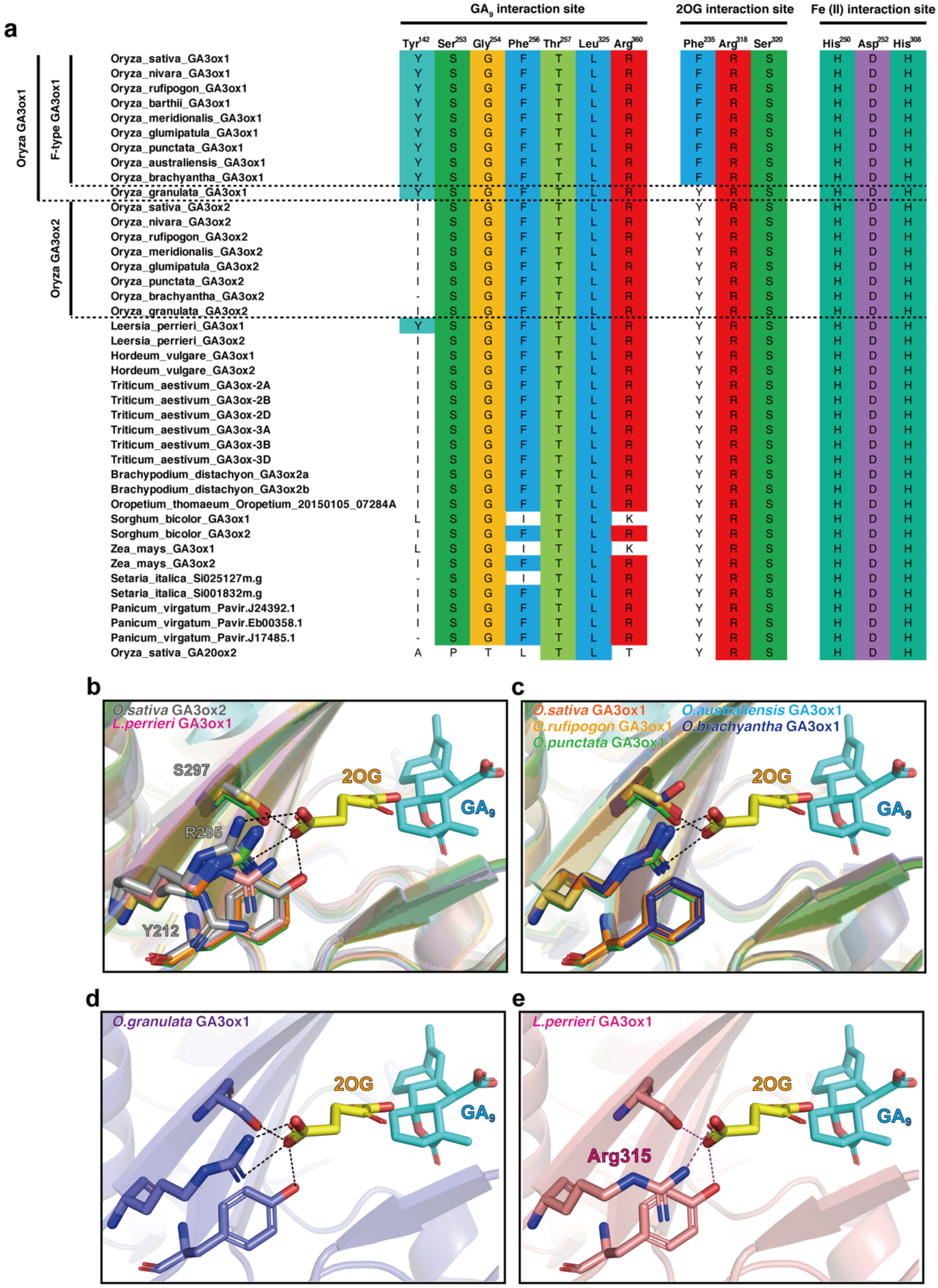

Supplementary Figure 10. Substrate interaction site of *Oryza* and *L. perrieri* GA3ox1. a) Alignment of GA_9, 2OG, and Fe(II) interacting site of Poaceae GA3ox orthologs. The residue numbers are those of OsGA3ox1 determined by the alignment analysis (Supplementary Figure 6). For *Oryza*

GA3ox1 orthologs, (co-) substrate interaction residues except Phe235 are conserved. **b-e)** Close-up view of the active site structure around 2OG in *Oryza* and *L. perrieri* GA3oxs as predicted by AlphaFold2 algorithms[14] (https://colab.research.google.com/github/sokrypton/ColabFold/blob/main/AlphaFold2.ipynb). Superimposed structures of *Oryza* and *L. perrieri* GA3ox1 and OsGA3ox2 (**b**) and *O. sativa*, *O. rufipogon* (AA), *O .punctata* (BB), *O. australiensis* (EE), *O. brachyantha* (FF) (**c**). The hydrogen bonds interactions of 2OG with residues of OsGA3ox2 (**b**) and *O. sativa* (**c**) are displayed. **d** and **e)** The structure of *O. granulata* (GG) (**d**) and *L. perrieri* (**e**). 2OG and GA_9, are shown with yellow and cyan sticks, respectively. Hydrogen bonds are indicated by dashed lines.

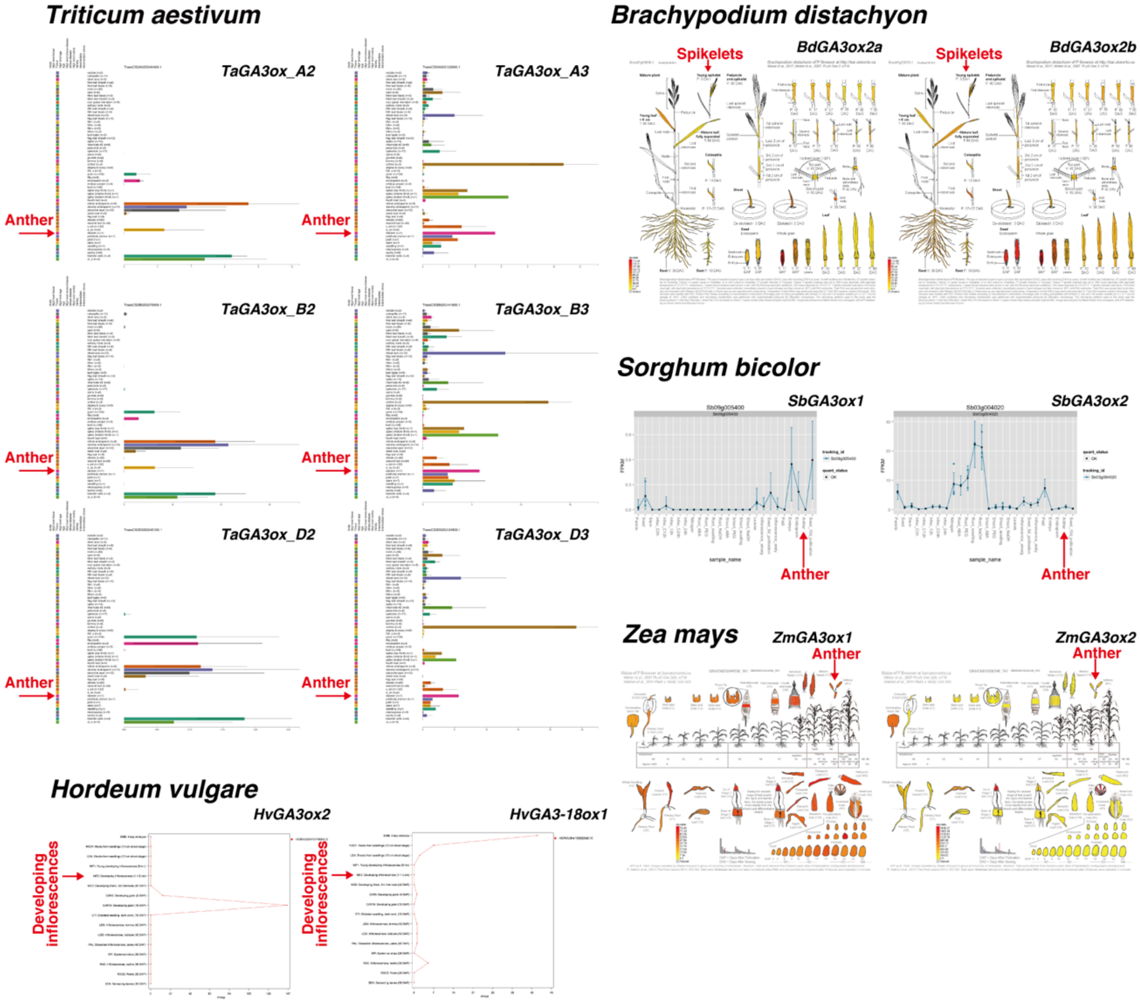

Supplementary Figure 11. Expression profiles of *GA3ox* orthologs in representative Poaceae plants. Tissue and developmental expression profile data of *GA3ox* orthologs in the Poaceae species *Triticum aestivum*, *Hordeum vulgare*, *Brachypodium distachyon*, *Sorghum bicolor,* and *Zea mays*. Expression profile data were obtained from the electronic fluorescent pictographs (eFP) browsers of the Brachypodium eFP Browser (*Brachypodium distachyon*) and Maize eFP Browser (*Zea mays*) (http://bar.utoronto.ca). Data for barley (*Hordeum vulgare*), wheat (*Triticum aestivum*), and sorghum (*Sorghum bicolor*) were obtained from PlaNet[15] (http://aranet.sbs.ntu.edu.sg/index.html), Wheat Expression Browser[16,17] (http://www.wheat-expression.com), and MOROKOSHI Sorghum transcriptome database[18], respectively. Signals of anthers or corresponding to them are indicated.

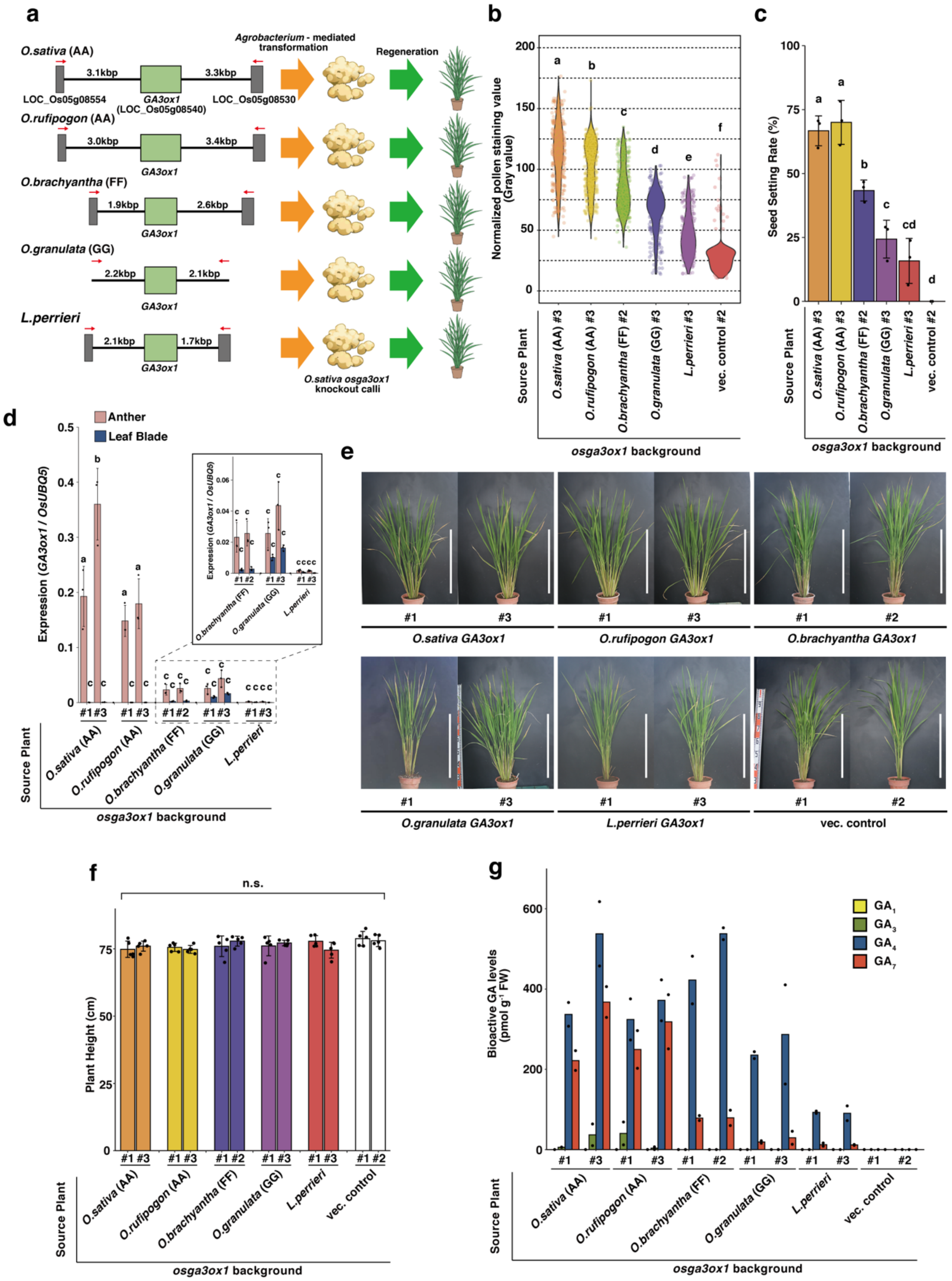

Supplementary Figure 12. Schematic of complementation test and phenotypic analysis. a) Schematic of transformation of *GA3ox1* orthologs of *Oryza* into *osga3ox1* mutant. *GA3ox1* orthologs (green); *GA3ox1* neighbor genes (grey); intergenic region (black line); primer for PCR (red arrow) in the left side of the diagram. **b)** Quantification of the staining value of KI-stained pollen grains of

transgenic plants and vector control. Error bars, s.d. n = 300, one-way ANOVA with Tukey's multiple comparison test. Different letters denote significant differences ($p < 0.05$). **c)** Seed setting rate of transgenic plants described in panel **a** and vector control. n = 3 panicles for each line; statistical analysis as mentioned in panel **b**. Figure 5**d** and **e**, and Supplementary Figure 12**b** and **c** represent the same experiment performed with different independent lines. **d)** qRT-PCR expression analysis of *GA3ox1* in anthers and leaf blades of each transformant. Error bars, s.d. n = 3, one-way ANOVA with Tukey's multiple comparisons test. Different letters denote significant differences ($p < 0.05$). **e)** The vegetative phenotype of each transformant. Bar = 50 cm. **e** Endogenous GA levels in anthers of transformant. **f)** Evaluation of plant heights of each transformant. Error bars, s.d. n = 5, one-way ANOVA. n.s.; not significant ($p > 0.05$). **g)** Endogenous GA levels in the anthers of transgenic plants and vector control. n = 2 per panicle. The line numbers used in **b-g** correspond to each other.

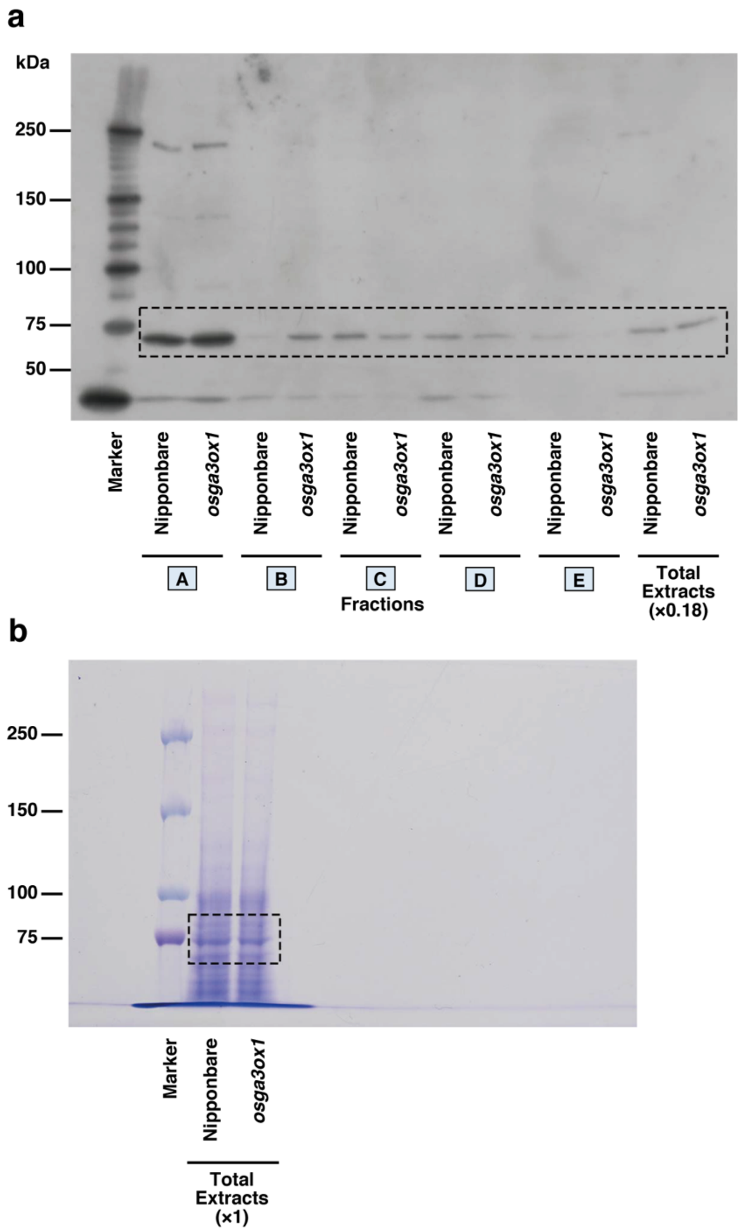

Supplementary Figure 13. Uncropped images of western blot and the CBB-stained gel. Full images of western blot (**a**) and CBB staining (**b**) that were depicted in part in Figure 2k. Dashed line represents the cropped area.

Supplementary References

1 Briand, C., Kozlov, S. V., Sonderegger, P. & Grütter, M. G. Crystal structure of neuroserpin: a neuronal serpin involved in a conformational disease. *FEBS Lett.* **505**, 18–22 (2001).

2 Otwinowski, Z. & Minor, W. Processing of X-ray diffraction data collected in oscillation mode. *Methods Enzymol.* **276**, 307–326 (1997).

3 McCoy, A. J., Grosse-Kunstleve, R. W., Storoni, L. C. & Read, R. J. Likelihood-enhanced fast translation functions. *Acta Crystallogr. D Biol. Crystallogr.* **61**, 458–464 (2005).

4 Sharp, A. M. *et al.* The active conformation of plasminogen activator inhibitor 1, a target for drugs to control fibrinolysis and cell adhesion. *Structure* **7**, 111–118 (1999).

5 Brünger, A. T. *et al*. Crystallography & NMR system: A new software suite for macromolecular structure determination. *Acta Crystallogr. D Biol. Crystallogr.* **54**, 905–921 (1998).

6 Bailey, S. The CCP4 suite: programs for protein crystallography. *Acta Crystallogr. D* **50**, 760–763 (1994).

7 Emsley, P. & Cowtan, K. Coot: model-building tools for molecular graphics. *Acta Crystallogr. D Biol. Crystallogr.* **60**, 2126–2132 (2004).

8 Takehara, S. *et al*. A common allosteric mechanism regulates homeostatic inactivation of auxin and gibberellin. *Nat. Commun.* **11**, 2143 (2020).

9 Katoh, K. & Standley, D. M. MAFFT multiple sequence alignment software version 7: improvements in performance and usability. *Mol. Biol. Evol.* version 7 **30**, 772–780 (2013).

10 Engelke, T., Hirsche, J. & Roitsch, T. Anther-specific carbohydrate supply and restoration of metabolically engineered male sterility. *J. Exp. Bot.* **61**, 2693–2706 (2010).

11 Ji, X. *et al.* Structure, evolution, and expression of the two invertase gene families of rice. *J. Mol. Evol.* **60**, 615–634 (2005).

12 Bouchenak-Khelladi, Y. *et al*. Large multi-gene phylogenetic trees of the grasses (Poaceae): progress towards complete tribal and generic level sampling. *Mol. Phylogenet. Evol.* **47**, 488–505 (2008).

13 Pearce, S. *et al*. Heterologous expression and transcript analysis of gibberellin

biosynthetic genes of grasses reveals novel functionality in the GA3ox family. *BMC Plant Biol.* **15** 130 (2015).

14 Jumper, J. *et al*. Highly accurate protein structure prediction with AlphaFold. *Nature* **596**, 583–589 (2021).

15 Mutwil, M. *et al*. PlaNet: combined sequence and expression comparisons across plant networks derived from seven species. *Plant Cell* **23**, 895–910 (2011).

16 Ramírez-González, R. *et al.* The transcriptional landscape of polyploid wheat. *Science* **361**, eaar6089 (2018).

17 Borrill, P., Ramirez-Gonzalez, R. & Uauy, C. expVIP: a customizable RNA-seq data analysis and visualization platform. *Plant Physiol.* **170**, 2172–2186 (2016).

18 Makita, Y. *et al.* MOROKOSHI: Transcriptome database in Sorghum bicolor. *Plant Cell Physiol.* **56**, e6 (2015).
