# Supplementary material for: Evolutionary alterations in gene expression and enzymatic activities of gibberellin 3-oxidase 1 in Oryza
Source: Commun Biol. 2022 Jan 19;5:67. doi: 10.1038/s42003-022-03008-5 (PMC8770518; doi:10.1038/s42003-022-03008-5)
Supplement: Supplementary file 3 — Description of Additional Supplementary Files [file 42003_2022_3008_MOESM3_ESM.pdf]

Description of Additional Supplementary Files

File name: Supplementary Video 1
Description: Time-lapse movie of Nipponbare flowering.

File name: Supplementary Video 2
Description: Time-lapse movie of *osga3ox1* flowering.

File name: Supplementary Data 1
Description: Source data underlying Figures in this study.

File name: Supplementary Data 2
Description: Statistical analysis of data performed in this study.

File name: Supplementary Data 3
Description: The sequenced data in this study.

File name: Supplementary Code 1
Description: ImageJ code to evaluate the normalized pollen staining value.
