# Supplementary material for: Evolutionary alterations in gene expression and enzymatic activities of gibberellin 3-oxidase 1 in Oryza
Source: Commun Biol. 2022 Jan 19;5:67. doi: 10.1038/s42003-022-03008-5 (PMC8770518; doi:10.1038/s42003-022-03008-5)
Supplement: Supplementary file 9 — Supplementary Code 1 [file 42003_2022_3008_MOESM9_ESM.pdf]

Supplementary Code 1: The script used for the analysis of Fig. 5b, d and Supplementary Fig. 12b. Please use the image of KI-stained pollen observed under a microscope as input to extract pollen regions and quantify their brightness.

```
/*
 * An imagej (fiji Version:2.0.0-rc-59/1.51n) script to quantify the degree of pollen staining.
 * 2020/10/06
 * Yosuke Toda.
 * ITbM, Nagoya Univ.
 * tyosuke@aquaseerser.com
 * https://totti0223.github.io/
 * https://www.phytometrics.jp/
 */

//GUI
#@ File (label = "Input directory", style = "directory") input

//Clear the Results Window and ROI info if present prior to analyis.
if (nResults > 0)
{
	run("Clear Results");
}

if (roiManager("count") > 0)
{
	roiManager("Deselect");
	roiManager("Delete");
}

//Create a subfolder named "outputs" in the same directory where the files to be analyzed are present.
output = input + File.separator+ "outputs";
File.makeDirectory(output);

//Main function
```

```
processFolder(input);

//Save csv to output directory using the Results window
selectWindow("Results");
saveAs("Measurements", output + File.separator + "Results.csv");

close("*");



//Scan image files and parse by processFile function
function processFolder(input) {
	list = getFileList(input);
	list = Array.sort(list);
	for (i = 0; i < list.length; i++) {

		s = list[i];
		dot = indexOf(s, ".");
		if (dot >= 0)
			s = substring(s, dot, lengthOf(list[i]));

		else
			s = 0;
		print(s);
		if (indexOf("..jpeg.jpg.JPEG.JPG.tiff.TIFF",s) > 0)
			processFile(input, output, list[i]);
	}
}

//Image analysis module
function processFile(input, output, file) {
	//Delete all roi first that are from the n-1 image. do not delete results table.
	if (roiManager("count") > 0)
	{
		roiManager("Deselect");
		roiManager("Delete");
	}
```

```
//open image
input_path = input + File.separator + file;
print("Processing: " + input_path);
open(input_path);
original=getTitle();

//get mask preprocessing
run("Duplicate...", " ");
mask=getTitle();
run("Gaussian Blur...", "sigma=2");
run("8-bit");
run("Subtract Background...", "rolling=40 light sliding");
run("Auto Threshold", "method=Default");

//depending on the image appearance, the below watershed process can be ignored by commenting out.
//run("Watershed");


//get candidate region
run("Analyze Particles...", "size=0-Infinity circularity=0.80-1.00 show=Overlay add");
roiManager("Set Color", "red");
roiManager("Set Line Width", 1);
run("Labels...", "color=blue font=12 show draw");

//save mask image
selectWindow(mask);
run("Hide Overlay");
maskpath = output + File.separator + file + "_1.jpg";
saveAs("Jpeg", maskpath);
close();

//get the b of hsb(hsv) for the degree of pollen staining.
selectWindow(original);
```

```
run("Show Overlay");
run("Duplicate...", " ");
hsb = getTitle();
run("HSB Stack");
run("Convert Stack to Images");
selectWindow("Hue");
close();
selectWindow("Saturation");
close();
selectWindow("Brightness");
brightness = getTitle();
run("Invert");
run("Show Overlay");

//append to results table with image file name column and pollen region id.
if (roiManager("count") > 0)
{
prevn = nResults;
roiManager("Measure");
print(prevn,nResults);
print("____");
//add filename to result table
//pollen id per image
k=0;
for (i = prevn; i < nResults; i++)
{
	setResult("filename", i, file);
	setResult("pollen_id", i, k);
	k+=1;
}
}

selectWindow(brightness);
close();

selectWindow(original);
```

```
        run("Show Overlay");
        run("Flatten");
        annpath = output + File.separator + file + "_2.jpg";
        saveAs("Jpeg", annpath);
        close();
        selectWindow(original);
        close();
}
```
