# Supplementary material for: Evolutionary alterations in gene expression and enzymatic activities of gibberellin 3-oxidase 1 in Oryza
Source: Commun Biol. 2022 Jan 19;5:67. doi: 10.1038/s42003-022-03008-5 (PMC8770518; doi:10.1038/s42003-022-03008-5)
Supplement: Supplementary file 10 — Reporting Summary [file 42003_2022_3008_MOESM10_ESM.pdf]

nature portfolio

Corresponding author(s): Miyako Ueguchi-Tanaka

Last updated by author(s): Dec 2, 2021

Reporting Summary

Nature Portfolio wishes to improve the reproducibility of the work that we publish. This form provides structure for consistency and transparency in reporting. For further information on Nature Portfolio policies, see our Editorial Policies and the Editorial Policy Checklist.

Statistics

For all statistical analyses, confirm that the following items are present in the figure legend, table legend, main text, or Methods section.

n/a	Confirmed	
☐	☒	The exact sample size (n) for each experimental group/condition, given as a discrete number and unit of measurement
☐	☒	A statement on whether measurements were taken from distinct samples or whether the same sample was measured repeatedly
☐	☒	The statistical test(s) used AND whether they are one- or two-sided<br>*Only common tests should be described solely by name; describe more complex techniques in the Methods section.*
☒	☐	A description of all covariates tested
☐	☒	A description of any assumptions or corrections, such as tests of normality and adjustment for multiple comparisons
☐	☒	A full description of the statistical parameters including central tendency (e.g. means) or other basic estimates (e.g. regression coefficient) AND variation (e.g. standard deviation) or associated estimates of uncertainty (e.g. confidence intervals)
☒	☐	For null hypothesis testing, the test statistic (e.g. F, t, r) with confidence intervals, effect sizes, degrees of freedom and P value noted<br>*Give P values as exact values whenever suitable.*
☒	☐	For Bayesian analysis, information on the choice of priors and Markov chain Monte Carlo settings
☒	☐	For hierarchical and complex designs, identification of the appropriate level for tests and full reporting of outcomes
☒	☐	Estimates of effect sizes (e.g. Cohen's d, Pearson's r), indicating how they were calculated

Our web collection on statistics for biologists contains articles on many of the points above.

Software and code

Policy information about availability of computer code

Data collection	X-ray data was collected from HKL2000.
Data analysis	Phenix version 1.17, WinCoot version 0.8.9.1, open-source PyMOL version 1.8.7, REFMAC5 version 7, R version 3.4, MAFFT version 7.3, MEGA version 10.

For manuscripts utilizing custom algorithms or software that are central to the research but not yet described in published literature, software must be made available to editors and reviewers. We strongly encourage code deposition in a community repository (e.g. GitHub). See the Nature Portfolio guidelines for submitting code & software for further information.

Data

Policy information about availability of data

All manuscripts must include a data availability statement. This statement should provide the following information, where applicable:

- Accession codes, unique identifiers, or web links for publicly available datasets
- A description of any restrictions on data availability
- For clinical datasets or third party data, please ensure that the statement adheres to our policy

The atomic coordinates and experimental data (codes 7EKD) have been deposited in the Protein Data Bank (www.wwpdb.org). All other data are available from the corresponding author on reasonable request.

Field-specific reporting

Please select the one below that is the best fit for your research. If you are not sure, read the appropriate sections before making your selection.

☒ Life sciences ☐ Behavioural & social sciences ☐ Ecological, evolutionary & environmental sciences

For a reference copy of the document with all sections, see nature.com/documents/nr-reporting-summary-flat.pdf

Life sciences study design

All studies must disclose on these points even when the disclosure is negative.

Sample size	Sample size number was determined empirically from past experience based on previous results obtained with these particular methods & approaches.
Data exclusions	No data were excluded from the analyses. Reflection outliers of crystallographic dataset were automatically excluded using established programs and criteria.
Replication	Three or more replicates were taken for the measurements, all attempts at replication were successful.
Randomization	Plants for each experiment were always chosen randomly.
Blinding	There has been no blinding.

Reporting for specific materials, systems and methods

We require information from authors about some types of materials, experimental systems and methods used in many studies. Here, indicate whether each material, system or method listed is relevant to your study. If you are not sure if a list item applies to your research, read the appropriate section before selecting a response.

Materials & experimental systems

n/a	Involved in the study	
☐	☒	Antibodies
☐	☒	Eukaryotic cell lines
☒	☐	Palaeontology and archaeology
☒	☐	Animals and other organisms
☒	☐	Human research participants
☒	☐	Clinical data
☒	☐	Dual use research of concern

Methods

n/a	Involved in the study	
☒	☐	ChIP-seq
☒	☐	Flow cytometry
☒	☐	MRI-based neuroimaging

Antibodies

Antibodies used	An antibody against OsCIN3 was prepared by immunizing rabbits with the synthetic peptide by Cosmo Bio Co., LTD. A goat anti-rabbit IgG-HRP secondary antibody (Invitrogen #65-6120) was used.
Validation	All antibodies were validated by the suppliers.

Eukaryotic cell lines

Policy information about cell lines

Cell line source(s)	Yeast (Saccharomyces cerevisiae) strain HA109 provided by Clontech Laboratories, Inc. was used.
Authentication	HA109 was verified by manufacture.
Mycoplasma contamination	The yeast strain was not tested for mycoplasma contamination.
Commonly misidentified lines (See ICLAC register)	No commonly misidentified lines are used in this study.
